# The Role of Palmar Cutaneous Branch Release in Enhancing Surgical Outcomes for Severe Carpal Tunnel Syndrome

**DOI:** 10.3390/jcm14072196

**Published:** 2025-03-24

**Authors:** Gokhan Sayer, Zeki Gunsoy, Fatih Golgelioglu, Omer Faruk Bayrakcioglu, Turan Bilge Kizkapan, Sener Ozboluk, Mustafa Dinc, Sinan Oguzkaya

**Affiliations:** 1 Department of Orthopedics and Traumatology, Bursa City Hospital, 16000 Bursa, Turkey; gkhnsyr38@gmail.com (G.S.); zekigunsoy@gmail.com (Z.G.); bayrakcioglu.omer@gmail.com (O.F.B.); senerozboluk@gmail.com (S.O.); drindianster@gmail.com (M.D.); sinanoguzkaya@hotmail.com (S.O.); 2 Department of Orthopedics and Traumatology, Faculty of Medicine, Yozgat Bozok University, 66100 Yozgat, Turkey; 3 Department of Orthopedics and Traumatology, School of Medicine, Bahcesehir Liv Hospital, Istinye University, 34000 Istanbul, Turkey; turanbilge.kizkapan@gmail.com

**Keywords:** carpal tunnel syndrome, median nerve, neurolysis, pain measurement, surgical decompression

## Abstract

**Background/Objectives:** Carpal tunnel syndrome (CTS) is the most common entrapment neuropathy and various surgical techniques are used for its treatment. Extended open carpal tunnel release (EOCTR) has been proposed for improved nerve decompression. This study compares the clinical and functional outcomes of open carpal tunnel release (OCTR) and EOCTR in severe CTS, hypothesizing superior functional outcomes and lower pain levels with EOCTR. **Methods:** This retrospective study included 53 patients (45 females, 8 males) with severe CTS confirmed by electromyography. Patients underwent either OCTR (n = 28) or EOCTR (n = 25) between January 2020 and February 2023. The EOCTR techinque involved additional neurolysis of the recurrent motor branch and palmar cutaneous branch of the median nerve. Functional outcomes were assessed using the Disabilities of the Arm, Shoulder, and Hand (DASH) questionnaire, the Boston Carpal Tunnel Syndrome Questionnaire (BCTQ), the Visual Analog Scale (VAS) for pain, and hand strength measurements. Complications and recovery parameters were also analyzed. **Results:** EOCTR resulted in significantly lower postoperative VAS scores (3.31 vs. 3.78, *p* < 0.001), DASH scores (16.54 vs. 20.68, *p* < 0.001), and BCTQ symptom scores (1.87 vs. 2.01, *p* < 0.001). No significant differences were found in grip strength (*p* = 0.52) or pinch strength (tip-to-tip: *p* = 0.54, lateral: *p* = 0.061, 3-point: *p* = 0.17). No major complications occurred, and pillar pain was similar in both groups (*p* = 0.82), resolving with conservative treatment. **Conclusions:** EOCTR with additional palmar cutaneous branch of the median nerve neurolysis may provide better short-term functional outcomes and lower pain levels compared to OCTR in severe CTS. Further prospective studies are needed to validate the long-term benefits and safety of this surgical approach.

## 1. Introduction

Carpal tunnel syndrome (CTS) is the most common entrapment neuropathy, with a significant impact on patients’ quality of life and daily functionality. It accounts for a substantial portion of work-related upper extremity disorders and frequently necessitates surgical intervention when conservative treatments fail [1]. While standard carpal tunnel release (CTR) effectively alleviates symptoms in most cases, some patients continue to experience postoperative complications such as persistent pain, sensory disturbances, and functional limitations. This variability in outcomes has led to ongoing discussions regarding the optimization of surgical techniques to improve clinical recovery [2,3].

The median nerve originates in the brachial plexus and courses through the arm without giving off significant branches. At the cubital fossa, it lies deep to the brachial artery and passes between the two heads of the pronator teres, where it gives rise to the anterior interosseous nerve (AIN). In the forearm, it travels between the flexor digitorum superficialis and profundus muscles, giving off the palmar cutaneous branch of the median nerve (PCBm) just proximal to the wrist. After passing through the carpal tunnel, it divides into the recurrent motor branch (RMB), which innervates the thenar muscles, and palmar digital nerves, which provide sensory innervation to the lateral fingers and palm. Given its anatomical course, incomplete decompression of certain branches, particularly the PCBm, has been implicated in persistent symptoms following surgery [4].

A major challenge in CTS surgery is ensuring adequate decompression of the median nerve while minimizing the risk of iatrogenic injury [5]. While open and mini-open techniques remain widely used, endoscopic carpal tunnel release (ECTR) has emerged as an alternative, with improved cosmetic results and faster recovery times [6,7]. However, concerns about inadequate release, particularly in cases with anatomical variations of the median nerve, have limited its widespread adoption. Specifically, the RMB and PCBm have been implicated in persistent postoperative symptoms, raising the question of whether additional surgical release of these structures may enhance clinical outcomes [8,9,10].

In recent years, ultrasound has been increasingly recognized as a valuable multiparametric tool for evaluating the median nerve in CTS diagnostics. High-resolution ultrasound allows for the assessment of morphometric and physical properties, microcirculation, and nerve movement, providing detailed anatomical and pathological insights. Although these advanced imaging modalities offer valuable diagnostic information, the most robust evidence for CTS evaluation remains the B-mode cross-sectional assessment of the median nerve cross-sectional area and the detection of structural abnormalities at the wrist. This approach helps refine diagnostic accuracy, ensuring appropriate surgical indications and treatment planning [11].

In our clinic experience, we have observed that a subset of patients with severe CTS continues to experience discomfort despite standard open carpal tunnel release (OCTR). We noted that in cases where additional neurolysis of the PCBm was performed, patients often reported superior symptom relief. This observation led us to investigate whether a more extensive surgical approach that includes targeted PCBm release could improve functional and pain-related outcomes.

The aim of this study was to compare the clinical and functional outcomes of patients who underwent extended OCTR versus standard OCTR for severe CTS. We hypothesized that extended OCTR, which includes additional PCBm release, would result in superior functional recovery and lower postoperative pain levels compared to the conventional approach.

## 2. Materials and Methods

### 2.1. Patient Selection

This retrospective case–control study was initiated following approval from the institutional ethics committee (approval date: 7 February 2024, approval number: 2024-1/7). The study included patients diagnosed with CTS who underwent surgical intervention at our clinic between 1 January 2020, and 1 February 2023. The diagnosis was confirmed by electromyography, conducted by an independent neurologist. The severity of CTS was classified based on the American Association of Neuromuscular and Electrodiagnostic Medicine (AANEM) criteria. The AANEM classification system categorizes CTS into mild, moderate, and severe stages based on electrodiagnostic findings. Mild CTS is characterized by delayed sensory conduction velocity while motor conduction remains intact. Moderate CTS presents with both sensory and motor conduction delays, with early signs of motor impairment. Severe CTS is defined by pronounced motor function loss, muscle atrophy, and significant sensory disturbances. This classification system is widely accepted as a standardized method for diagnosing and assessing CTS severity, guiding both treatment decisions and prognosis [12].

**Figure 1:**
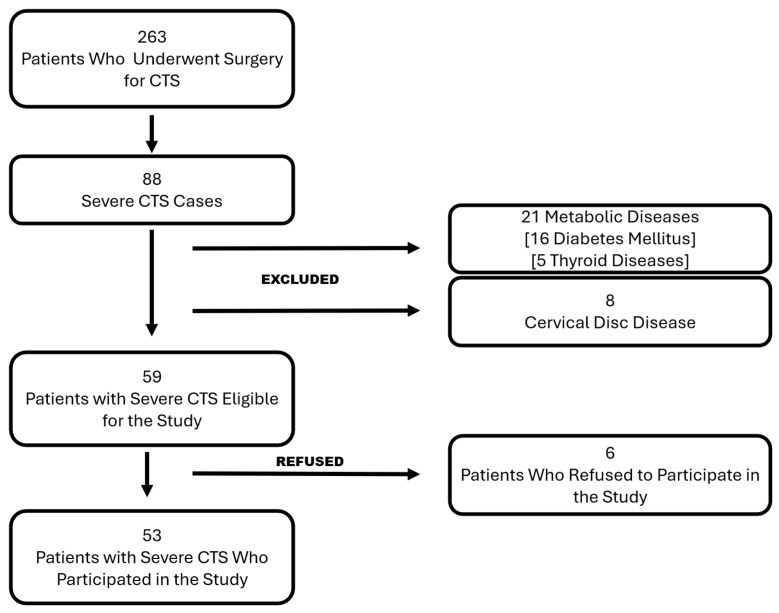
Selection process flow chart. CTS: Carpal tunnel syndrome.

Patients eligible for inclusion met the following criteria: they were 18 years or older, had a confirmed CTS diagnosis requiring surgical intervention, and had undergone either the standard OCTR or the extended open carpal tunnel release (EOCTR). Furthermore, a minimum of one year of postoperative follow-up was required to assess long-term outcomes. Patients with a history of cervical disc pathology, metabolic diseases known to cause peripheral neuropathy such as diabetes mellitus or hypothyroidism, or other neurological conditions leading to motor or sensory impairment were excluded. Patients with a history of previous fracture or surgical treatment of the ipsilateral forearm, wrist, or hand were also excluded from the study. Additionally, patients who had undergone previous CTS surgery or required revision surgery during the follow-up period were not included in the study. The selection process is documented and summarized in Figure 1.

### 2.2. Surgical Techniques

The surgeries were performed by three experienced orthopedic and traumatology surgeons, each with at least five years of experience in the hand. The choice of surgical method was based on the surgeon’s preference. One surgeon exclusively performed the extended approach, while the other two used the classical approach. All surgical procedures were conducted under either axillary block or general anesthesia. A pneumatic tourniquet was applied in all cases, with the pressure set 70 mmHg above the preoperative systolic blood pressure.

**Figure 2:**
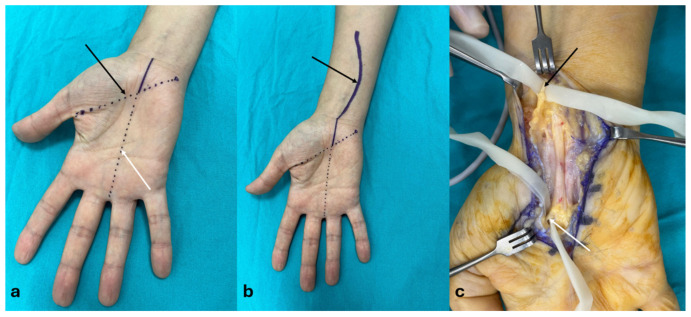
(**a**) The classical approach incision (purple line): The black arrow indicates the Kaplan’s line (purple dashed line) and the white arrow indicates the radial side of the 4th finger (purple dashed line). (**b**) Extended approach incision (purple line): The Black arrow indicates the proximally extending lazy S incision, which begins at the Kaplan’s line (purple dashed line) at the radial side of the 4th finger (purple dashed line). (**c**) Extended approach: Release of the palmar cutaneous branch of the median nerve (black arrow) proximally and the recurrent motor branch (white arrow) distally.

For the classical approach (group OCTR), a 3–4 cm curvilinear incision was made along Kaplan’s cardinal line (KCL) and the radial border of the fourth finger. The transverse carpal ligament (TCL) was identified and incised after dissecting through the subcutaneous fat and palmar fascia (Figure 2a). For the extended approach (group EOCTR), the procedure was performed using 3.5× loupe magnification. A longitudinal incision was made at the intersection of the line passing through the middle of the 3–4 intermetacarpal space and KCL, extending to the flexor crease, and then continued distally with a lazy S incision, reaching the third of the forearm (Figure 2b). After exposing and dividing the TCL, the sheaths of the palmaris longus and flexor carpi radialis tendons were incised. The median nerve was carefully explored beneath the palmaris longus tendon, freed from surrounding adherent tissues, and subjected to external neurolysis. The RMB was traced up to its entry into the thenar muscle and underwent neurolysis. Additionally, the PCBm of the median nerve was explored, followed by distal external neurolysis. The wound was closed using mattress sutures (Figure 2c).

### 2.3. Outcome Measures

Patient demographic data, including age, gender, affected side, dominant hand, and body mass index (BMI), were collected from medical records. Preoperative assessments included the Disabilities of the Arm, Shoulder, and Hand (DASH) questionnaire and the Boston Carpal Tunnel Syndrome Questionnaire (BCTQ) [13,14]. Patients were given detailed instructions before completing these questionnaires. The BCTQ consists of two subscales: the symptom severity scale (SSS), with 11 items; and the functional scale (FS), with 8 items; each scored on a Likert scale ranging from 1, indicating no symptoms, to 5, indicating very severe symptoms [15]. Pain levels were assessed using the Visual Analog Scale (VAS), from 0 to 10.

At the final follow-up, patients completed the DASH and BCTQ questionnaires again, and their pain was reassessed using the VAS. Additionally, grip and pinch strength were measured with a hand-held dynamometer and pinch meter.

Postoperative complications, including infection, hematoma, paresthesia, and pillar pain, were recorded. Pillar pain was evaluated using the Table Test [16], where patients placed their weight on a table with locked elbows in extension. Pain elicited during this maneuver was considered indicative of pillar pain.

### 2.4. Statistical Analysis

Descriptive statistics, including mean, median, frequency, minimum, maximum, and standard deviation (SD), were used to summarize the data. The Shapiro–Wilk test was applied to assess the normality of the variables. For comparisons between the two independent groups, the Mann–Whitney U test was used for continuous variables, while the chi-square test was employed for categorical variables. A paired-samples t-test was conducted for the analysis of dependent variables. Statistical significance was determined by a *p*-value of less than 0.05. All statistical analyses were performed using IBM SPSS for Windows, version 26 (IBM Corp., Armonk, NY, USA).

## 3. Results

This study included 53 patients (45 females, 8 males) with a mean age of 50.4 ± 7.7 years. There were no significant differences between the groups in terms of demographic characteristics or follow-up duration (*p* > 0.05). Additional demographic details are presented in Table 1.

Significant differences were found between the groups in postoperative VAS (3.3 ± 0.3 vs. 3.8 ± 0.2, *p* < 0.001), DASH (16.5 ± 1.6 vs. 20.7 ± 2.7, *p* < 0.001), and BCTQ-SS scores (1.9 ± 0.1 vs. 2 ± 0.1, *p* < 0.001) (Table 2).

No patients required revision surgery during follow-up. One patient in the EOCTR group developed signs of a superficial infection, which resolved after 10 days of oral antibiotic therapy. There were no indications of vascular or nerve injury in either group.

Pillar pain was reported in five patients (EOCTR: 3, OCTR: 2; *p* = 0.82). Triamcinolone injections were administered at the 9-month follow-up, and symptoms had resolved in all patients by the final examination.

## 4. Discussion

The most important finding of the current study was that the EOCTR technique, which includes additional PCBm neurolysis, was associated with significantly lower postoperative pain levels and superior functional outcomes compared to the standard OCTR in patients with severe CTS. This result strongly supports the hypothesis that incomplete decompression of the median nerve, particularly involving the PCBm, may contribute to persistent symptoms in CTS patients. The improved postoperative DASH, BCTQ-SS, and VAS scores in the EOCTR group highlight the potential role of PCBm neurolysis in enhancing sensory recovery and reducing residual pain. These findings underscore the importance of addressing anatomical variations of the median nerve and its branches during CTS surgery to optimize clinical outcomes.

Previous studies comparing mini-open and extended approaches, such as those by Akkurt et al. and Murthy et al. [17,18], did not find significant differences in clinical or functional outcomes. However, the surgical techniques used in these studies differed from our approach. Akkurt et al. performed an incision extending only 1 cm beyond the KCL and concluded the procedure with neurolysis after TCL release. Murthy et al. extended the incision 2–4 cm proximal to the wrist crease but did not perform internal neurolysis. In contrast, our extended approach included a more comprehensive release involving PCBm neurolysis, which may explain the superior pain relief and functional improvement observed in our study.

Although the PCBm does not pass through the carpal tunnel itself, it courses within its own long tunnel at the level of the transverse carpal ligament, positioned between the carpal ligament and the flexor retinaculum [19]. Elevated pressure within the carpal tunnel may lead to secondary morphological changes in the PCBm [20]. In severe CTS cases, prolonged exposure to high intra-tunnel pressure can affect the PCBm, leading to ischemic or compressive neuropathy. Performing neurolysis on this branch may contribute to reducing local pressure, potentially improving sensory function and pain relief in affected patients. This mechanism provides a plausible explanation for why decompression of the PCBm, despite its extrinsic location, may enhance postoperative outcomes in severe CTS cases.

The PCBm is a distal collateral branch that provides sensory innervation to the proximal palm and thenar eminence [21]. Traditionally, it was believed that the PCBm was not involved in CTS, as it does not traverse the carpal tunnel [22]. However, electrophysiological studies conducted by Uluc et al. demonstrated abnormal PCBm nerve conduction in 56% of CTS patients [19]. Similarly, Rathakrishnan et al. [22] reported significantly impaired PCBm conduction in CTS patients compared to controls. Furthermore, Jeong et al. [20], utilizing high-resolution ultrasound in a study involving healthy volunteers and CTS patients, observed a significantly increased PCBm cross-sectional area in severe CTS cases, suggesting the involvement of this branch in the disease process. Also, it was reported that PCBm entrapment can be seen in CTS [9,23]. Based on our results, we can speculate that an unreleased PCBm may reduce patient satisfaction from the surgery.

Although ECTR has been associated with faster recovery and better cosmetic outcomes, long-term studies have shown similar functional results between ECTR and OCTR [5,24,25]. Aslani et al. [26] compared OCTR, mini-open carpal tunnel release (mOCTR), and ECTR, reporting better early outcomes with mOCTR and ECTR, but no significant differences beyond the fourth postoperative month. Similarly, Yücetaş et al. [27] found comparable functional outcomes between mOCTR and OCTR at six months. In our study, no significant differences were observed between the EOCTR and OCTR groups regarding grip strength, pinch strength, and BCTQ functional scale (BCTQ-FS). However, the significant differences in VAS and BCTQ-SSS scores suggest that while motor function recovery was similar between groups, sensory outcomes improved more in the EOCTR group. This could indicate that RMB neurolysis does not significantly impact motor recovery, whereas PCBm release plays a crucial role in sensory improvement.

Postoperative complications in CTS surgery can include nerve injuries, vascular damage, and tendon injuries [28]. The risk of nerve injury is reportedly higher with ECTR than with OCTR, although most injuries are transient [29]. Given the anatomical variations in the median nerve and its branches, as well as the proximity of the TCL to adjacent structures, all techniques carry some risk of iatrogenic injury [30]. Extended OCTR is often considered safer in addressing anatomical variations and preventing complications [31]. In our study, no vascular or nerve injuries were observed in either group. One patient in the EOCTR group developed a superficial infection, which resolved with oral antibiotics. Pillar pain, a common postoperative complaint, was reported in five patients (EOCTR: 3, OCTR: 2), with symptoms successfully managed using triamcinolone injections by the nine-month follow-up.

This study is the first to evaluate the impact of additional PCBm neurolysis in the surgical management of severe CTS. In this regard, we believe it addresses an important gap in the literature. These findings have significant clinical implications. Surgeons should carefully consider the role of the PCBm in persistent postoperative symptoms when planning surgical treatment for severe CTS. Recognizing the potential contribution of this branch to residual pain and sensory disturbances may help refine surgical strategies and optimize patient outcomes. Future research should focus on exploring the potential benefits of intraoperative electrophysiological monitoring or imaging modalities to better assess PCBm involvement in CTS pathology. Additionally, comparative studies evaluating the long-term outcomes of EOCTR versus other minimally invasive techniques could provide further insight into the effectiveness of different surgical approaches. Prospective, multicenter trials with larger patient cohorts would strengthen the validity of these findings and guide the development of standardized protocols for CTS surgery, ultimately improving functional recovery and reducing postoperative complications.

This study has several limitations. First, as a retrospective analysis, it is subject to selection bias and limited by the availability of documented data. Second, the relatively small sample size may restrict the generalizability of the findings, and future studies with larger cohorts are warranted. Third, although all procedures were performed by experienced hand surgeons, the choice of surgical approach was based on the individual surgeon’s preference, which could introduce an operator-dependent bias. However, all surgeons followed a standardized surgical protocol, and the primary difference between techniques was the additional neurolysis of the palmar cutaneous branch in the EOCTR group. Additionally, all participating surgeons had more than five years of experience in hand surgery, which helps mitigate variability in surgical skill. Fourth, follow-up was limited to one year, and longer-term outcomes remain unknown. Finally, electrophysiological assessments of PCBm involvement were not included, which could have provided further insights into the role of this branch in persistent CTS symptoms. Another limitation of our study is that multiple statistical comparisons were performed without applying correction methods. However, since the analyses were conducted based on predefined hypotheses focusing on key functional and pain-related outcomes, such corrections were not deemed necessary. Nevertheless, we acknowledge the potential risk of increased type I error, and our results should be interpreted accordingly.

## 5. Conclusions

This study suggests that EOCTR with additional PCBm neurolysis may provide better postoperative pain relief and functional outcomes compared to standard OCTR in severe CTS cases. The significantly lower VAS and BCTQ-SSS scores in the EOCTR group indicate that persistent postoperative symptoms might be linked to incomplete decompression of the median nerve, particularly involving the PCBm. However, given the small sample size, lack of statistical corrections, and potential variations in surgical techniques among operators, our conclusions should be interpreted with caution. While the findings support the hypothesis that EOCTR could enhance clinical outcomes, further large-scale, prospective studies with standardized surgical protocols and long-term follow-up are necessary to confirm the effectiveness and safety of this approach. Future research should also explore whether preoperative imaging or electrophysiological assessments can help identify patients who would benefit most from PCBm neurolysis, as well as assess the durability of clinical improvements over an extended period.

## Figures and Tables

**Table 1:** Demographic and clinical characteristics of patients undergoing extended open carpal tunnel release and open carpal tunnel release.

Variable	Entire Study Population (n = 53)	Group EOCTR (n = 24)	Group OCTR (n = 29)	*p*
Age, year (mean ± SD)	50.4 ± 7.7	50.3 ± 7.3	50.4 ± 8.1	0.94
Gender, n (%)				
Female	45 (85)	21 (88)	24 (83)	0.71
Male	8 (15)	3 (12)	5 (17)	
Side, n (%)				
Right	45 (85)	19 (79)	26 (90)	0.44
Left	8 (15)	5 (21)	3 (10)	
Dominance status, n (%)				
Dominant	41 (77)	18 (75)	23 (79)	0.75
Non-dominant	12 (23)	6 (25)	6 (22)	
BMI (mean ± SD)	26.5 ± 2.7	27.2 ± 2.6	26 ± 2.7	0.12
Follow-up, months (mean ± SD)	22 ± 5.0	22 ± 5.4	21 ± 4.7	0.42

EOCTR: extended open carpal tunnel release, OCTR: open carpal tunnel release, SD: standard deviation, BMI: body mass index.

**Table 2:** Comparison of clinical and functional outcomes between extended open carpal tunnel release and open carpal tunnel release.

Parameter	Group EOCR (Mean ± SD)	Group OCR (Mean ± SD)	*p*-Value
Preoperative VAS	7.5 ± 0.4	7.8 ± 0.6	0.076
Postoperative VAS	3.3 ± 0.3	3.8 ± 0.2	<0.001
Preoperative DASH	47.2 ± 2.9	47.7 ± 4.1	0.802
Postoperative DASH	16.5 ± 1.6	20.7 ± 2.7	<0.001
Preoperative BCTQ-SSS	3.9 ± 0.2	3.9 ± 0.2	0.878
Postoperative BCTQ-SSS	1.9 ± 0.1	2 ± 0.1	<0.001
Preoperative BCTQ-FS	3.9 ± 0.3	3.9 ± 0.2	0.971
Postoperative BCTQ-FS	2 ± 0.1	2.1 ± 0.1	0.058
Grip Strength (kg)	22.7 ± 1.4	22.4 ± 1.6	0.520
Pinch Strength (kg)—Tip-to-Tip	2.4 ± 0.3	2.3 ± 0.2	0.540
Pinch Strength (kg)—Lateral	3.1 ± 0.4	3 ± 0.2	0.061
Pinch Strength (kg)—3-Point	3 ± 0.3	2.9 ± 0.1	0.170

EOCR: extended open carpal tunnel release, OCR: open carpal tunnel release, SD: standard deviation, VAS: Visual Analog Scale, DASH: Disabilities of the Arm, Shoulder, and Hand Questionnaire, BCTQ-SSS: Boston Carpal Tunnel Syndrome Questionnaire—Symptom Severity Scale, BCTQ-FS: Boston Carpal Tunnel Syndrome Questionnaire—Functional Scale.

## Data Availability

The data supporting the reported results in this study were obtained from the Web of Science Core Collection (WoSCC, Clarivate Analytics). These data are publicly available to users with access to the database. No new datasets were generated during the study.

## Abbreviations

CTS	Carpal tunnel syndrome
CTR	Carpal tunnel release
OCTR	Open carpal tunnel release
ECTR	Endoscopic carpal tunnel release
RMB	Recurrent motor branch
PCBm	Palmar cutaneous branch of the median nerve
EOCTR	Extended open carpal tunnel release
AANEM	American Association of Neuromuscular and Electrodiagnostic Medicine
TCL	Transverse carpal ligament
KCL	Kaplan’s cardinal line
DASH	Disabilities of the Arm, Shoulder, and Hand Questionnaire
BCTQ	Boston Carpal Tunnel Syndrome Questionnaire
SSS	Symptom severity scale
FS	Functional scale
VAS	Visual Analog Scale
mOCTR	Mini-open carpal tunnel release
